# Supplementary material for: Comparing the Indian Autism Screening Questionnaire (IASQ) and the Indian Scale for Assessment of Autism (ISAA) with the Childhood Autism Rating Scale–Second Edition (CARS2) in Indian settings
Source: PLoS One. 2022 Sep 19;17(9):e0273780. doi: 10.1371/journal.pone.0273780 (PMC9484635; doi:10.1371/journal.pone.0273780)
Supplement: S1 Table — (DOCX) [file pone.0273780.s001.docx]

Supplementary Table 1a: Sensitivity and specificity of IASQ with ISAA (n=124)

| IASQ | National Institute for the Empowerment of Persons with Intellectual Disability (NIEPID) Sample (n=124) | | | | |
| --- | --- | --- | --- | --- | --- |
| Cut off | Sensitivity | Specificity | Likelihood Ratio LR+ | NPV | PPV |
| 1 | 0.97 | 0.38 | 1.56 | 0.92 | 0.64 |
| 2 | 0.97 | 0.71 | 3.31 | 0.95 | 0.79 |
| 3 | 0.95 | 82.76 | 5.54 | 0.94 | 0.86 |
| 4 | 0.94 | 87.93 | 7.78 | 0.88 | 0.90 |
| 5 | 92.30 | 89.83 | 9.08 | 0.91 | 0.92 |
| 6 | 84.85 | 94.83 | 16.40 | 0.85 | 0.95 |
| 7 | 78.79 | 94.83 | 15.23 | 0.80 | 0.95 |
| 8 | 59.09 | 100.00 | Infinity | 0.68 | 1.00 |
| 9 | 34.85 | 100.00 | Infinity | 0.57 | 1.00 |
| 10 | 4.55 | 100.00 | Infinity | 0.48 | 1.00 |
