# Supplementary material for: Comparing the Indian Autism Screening Questionnaire (IASQ) and the Indian Scale for Assessment of Autism (ISAA) with the Childhood Autism Rating Scale–Second Edition (CARS2) in Indian settings
Source: PLoS One. 2022 Sep 19;17(9):e0273780. doi: 10.1371/journal.pone.0273780 (PMC9484635; doi:10.1371/journal.pone.0273780)
Supplement: S2 Table — (DOCX) [file pone.0273780.s002.docx]

Supplementary Table 1b: Sensitivity and specificity of IASQ with ISAA (n=157)

| IASQ | CGHS Housing Units (n=157) | | | | |
| --- | --- | --- | --- | --- | --- |
| Cut off | Sensitivity | Specificity | Likelihood Ratio LR+ | NPV | PPV |
| 1 | 100.00 | 97.44 | 39 | 1 | 0.2 |
| 2 | 100.00 | 99.60 | 156 | 1 | 0.5 |
| 3 | 100.00 | 100.00 | -Infinity | 1 | 1 |
| 4 | 100.00 | 100.00 | -Infinity | 1 | 1 |
| 5 | 100.00 | 100.00 | -Infinity | 1 | 1 |
| 6 | 100.00 | 100.00 | -Infinity | 1 | 1 |
| 7 | 100.00 | 100.00 | -Infinity | 1 | 1 |
| 8 | 100.00 | 100.00 | -Infinity | 1 | 1 |
| 9 | 100.00 | 100.00 | -Infinity | 1 | 1 |
| 10 | 100.00 | 100.00 | -Infinity | 0.99 | 1 |
