# Supplementary material for: Comparing the Indian Autism Screening Questionnaire (IASQ) and the Indian Scale for Assessment of Autism (ISAA) with the Childhood Autism Rating Scale–Second Edition (CARS2) in Indian settings
Source: PLoS One. 2022 Sep 19;17(9):e0273780. doi: 10.1371/journal.pone.0273780 (PMC9484635; doi:10.1371/journal.pone.0273780)
Supplement: Attachment — Submitted filename: iasq cars 2 Reply to reviewers comments25052022.docx [file pone.0273780.s003.docx]

Reply to reviewers’ and editorial comments:

Editorial comments

Comment 1. Please ensure that your manuscript meets PLOS ONE's style requirements, including those for file naming. The PLOS ONE style templates can be found at

<https://journals.plos.org/plosone/s/file?id=wjVg/PLOSOne_formatting_sample_main_body.pdf> and

<https://journals.plos.org/plosone/s/file?id=ba62/PLOSOne_formatting_sample_title_authors_affiliations.pdf>.

Reply1: Thank you. We have checked these and made the necessary changes.

Comment 2. We note that the grant information you provided in the ‘Funding Information’ and ‘Financial Disclosure’ sections do not match. When you resubmit, please ensure that you provide the correct grant numbers for the awards you received for your study in the ‘Funding Information’ section.

Reply2: Thank you for the guidance. We have changed the funding information as under:

Funding: This research was funded by the Indian Council of Medical Research Task Force for Capacity Building for National Mental Health Programme (File No No. 5/4-4/151/M/2017/NCD-I) and the Fogarty International Centre, NIH (‘Cross-Fertilized Research Training for New Investigators in India and Egypt’, D43 TW009114 Fogarty International Centre, NIH and HMSC File No. Indo-Foreign/35/M/2012-NCD-1).The content of this manuscript is solely the responsibility of the authors and does not necessarily represent the official views of Fogarty International Centre, NIH (Cross-Fertilized Research Training for New Investigators in India and Egypt’ (D43 TW009114) or ICMR had no role in the design and conduct of the study; collection, management, analysis, and interpretation of the data; preparation, review, or approval of the manuscript; and decision to submit the manuscript for publication

Comment 3. Thank you for stating the following in the Acknowledgments Section of your manuscript:

“We thank Dr. Soumya Swaminathan (then Secretary, Dept. of Health Research, DHR), Dr. Balram Bhargav (Secretary DHR), and Dr. Harpreet Singh, ICMR. We thank the faculty of ‘Cross-Fertilized Research Training for New Investigators in India and Egypt’ (D43 TW009114, HMSC File No. Indo-Foreign/35/M/2012-NCD-1, funded by Fogarty International Centre, NIH).   We thank the National Coordinating Unit for logistic support and the ICMR Data Management Unit for designing the database.  The content of this manuscript is solely the responsibility of the authors and does not necessarily represent the official views of NIH or ICMR. NIH and ICMR had no role in the design and conduct of the study; collection, management, analysis, and interpretation of the data; preparation, review, or approval of the manuscript; and decision to submit the manuscript for publication.”

We note that you have provided funding information that is not currently declared in your Funding Statement. However, funding information should not appear in the Acknowledgments section or other areas of your manuscript. We will only publish funding information present in the Funding Statement section of the online submission form.

Please remove any funding-related text from the manuscript and let us know how you would like to update your Funding Statement. Currently, your Funding Statement reads as follows:

“The funders had no role in study design, data collection and analysis, decision to publish, or preparation of the manuscript.”

Please include your amended statements within your cover letter; we will change the online submission form on your behalf.

Reply3: Thank you for clarifying this. We have deleted the funding information in the manuscript.

The funding statement needs to be changed as follows:

This research was funded by the Indian Council of Medical Research Task Force for Capacity Building for National Mental Health Programme (File No No. 5/4-4/151/M/2017/NCD-I) and the Fogarty International Centre, NIH (‘Cross-Fertilized Research Training for New Investigators in India and Egypt’, D43 TW009114 Fogarty International Centre, NIH and HMSC File No. Indo-Foreign/35/M/2012-NCD-1The content of this manuscript is solely the responsibility of the authors and does not necessarily represent the official views of Fogarty International Centre, NIH (Cross-Fertilized Research Training for New Investigators in India and Egypt’ (D43 TW009114) or ICMR had no role in the design and conduct of the study; collection, management, analysis, and interpretation of the data; preparation, review, or approval of the manuscript; and decision to submit the manuscript for publication.

Comment 4. Please **amend your list of authors on the manuscript to ensure that each author is linked to an affiliation. Authors’ affiliations should reflect the institution where the work was done (if authors moved subsequently, you can also list the new affiliation stating “current affiliation:….” as necessary)**.

Reply 4: All authors are linked to affiliation

Comment 5. Please include a caption for figure 1.

Reply 5: I have included the caption of all figures in the manuscript.

Comment 6: Please review your reference list to ensure that it is complete and correct. If you have cited papers that have been retracted, please include the rationale for doing so in the manuscript text, or remove these references and replace them with relevant current references. Any changes to the reference list should be mentioned in the rebuttal letter that accompanies your revised manuscript. If you need to cite a retracted article, indicate the article’s retracted status in the References list and also include a citation and full reference for the retraction notice.

[Note: HTML mark-up is below. Please do not edit.]

Reply 6: Reference list is checked. It is complete and is according to PLOS ONE's style.

**Reviewers' comments:**

Reviewer's Responses to Questions

**Comments to the Author**

1. Is the manuscript technically sound, and do the data support the conclusions?

The manuscript must describe a technically sound piece of scientific research with data that supports the conclusions. Experiments must have been conducted rigorously, with appropriate controls, replication, and sample sizes. The conclusions must be drawn appropriately based on the data presented.

Reviewer #1: Yes

2. Has the statistical analysis been performed appropriately and rigorously?

Reviewer #1: Yes

3. Have the authors made all data underlying the findings in their manuscript fully available?

The [PLOS Data policy](http://www.plosone.org/static/policies.action#sharing) requires authors to make all data underlying the findings described in their manuscript fully available without restriction, with rare exception (please refer to the Data Availability Statement in the manuscript PDF file). The data should be provided as part of the manuscript or its supporting information, or deposited to a public repository. For example, in addition to summary statistics, the data points behind means, medians and variance measures should be available. If there are restrictions on publicly sharing data—e.g. participant privacy or use of data from a third party—those must be specified.

Reviewer #1: Yes

4. Is the manuscript presented in an intelligible fashion and written in standard English?

PLOS ONE does not copyedit accepted manuscripts, so the language in submitted articles must be clear, correct, and unambiguous. Any typographical or grammatical errors should be corrected at revision, so please note any specific errors here.

Reviewer #1: Yes

Reply: Thank you very much for this endorsement, respected reviewer.

5. Review Comments to the Author

Reviewer #1:

Query 1: This paper dealing with the psychometric properties of two autism screening scales validated against the CARS 2 has several strengths and one or two weaknesses. The latter concerns the English language which could be improved and that there is one omission when it comes to the analytic plan.

The research procedures seem rigorous based on the descriptions and the data analyses are performed in an expert way. The concordant validity and discriminant validity are well described and shows adequate properties.

Reply 1: Thank you very much for the appreciation and encouraging remarks. We have rechecked the manuscript for English language and simplified text to the extent possible.

Query 2.There data when it comes to psychometrics: i.e. sensitivity, specificity, PPV and NPV as well as the ROC-analysis are to the point. The omission is, I think that the likelihood ratio (diagnostic likelihood ratio (DLR) could be used in a clearer way and not just represented in a table. This is important when the diagnostic task involves a low prevalence disorder such a as autism. The reason I'd like to highlight it is that using Bayesian thinking even reasonably high DLR that are shown based on a ISAAQ cut-off of 2 or 3 points (roughly 11 and 20 OR) render quite a weak signal as what to do with a positive cut-off. When the scales are used in a screening situation where the base rate of autism is close to 1%, the odds for an autism diagnosis does not raise higher than 20 respectively 30% from a score above the cut-off. These odds are roughly calulated based on a nomogram as described by Youngstrom (e.g., 1. Youngstrom E. Future Directions in Psychological Assessment: Combining Evidence-Based Medicine Innovations with Psychology's Historical Strengths to Enhance Utility. Journal of Clinical Child and Adolescent Psychology. 2013;42(1):139-59.

Thus, I would like authors to argue in a more explicit way on what scores should be used and what needs to be done following a positive screening outcome.

As a summary, however, I like the paper and I feel its utility would be enhanced if my suggestions for improvements are used.

Reply 2:. We thank the reviewer for liking the manuscript. The reviewer correctly points out the non-linear nature of the diagnostic likelihood ratio or DLR (denoted by LR+ in the manuscript). While this is a known drawback of this metric, it provides the best measure of the discriminative power of the test by summarizing both its sensitivity and specificity.  Furthermore, unlike sensitivity or specificity, DLR remains unaffected by the prevalence of the disease.  We have therefore added clarification in the manuscript to aid the reader in interpreting the DLR and its utility in clinical decision making.

We have added text to explain this matter in various sections as follows:

Methods:

Test psychometrics:

…. Likelihood ratios provide the best guide to clinicians to determine whether a diagnostic test usefully changes the odds that a disease exists.  By convention, a positive diagnostic likelihood ratio (LR+) between 1 to 5 is associated with a small increase in post-test odds whereas values between 5 and 10 with a moderate increase and values above 10 with a large increase in post-test odds. Both ranges, 5 to 10 and >10 are considered acceptable for clinical decision making. To further examine the effects that these LR+s may have on the likelihood of disease a Fagan’s Nomogram may be used after first converting the pre-test odds to a pre-test probability and then drawing a straight line from the pre-test probability through the LR+ to the corresponding post-test probability [25, 26]. Alternatively, interactive online calculators, for example the diagnostic test calculator available at the University of Illinois, Chicago, USA  ([http://araw.mede.uic.edu/cgi-bin/testcalc.pl](https://nam12.safelinks.protection.outlook.com/?url=http%3A%2F%2Faraw.mede.uic.edu%2Fcgi-bin%2Ftestcalc.pl&data=05%7C01%7Cssi%40pitt.edu%7Ce62d393889c346f565b108da37ece42e%7C9ef9f489e0a04eeb87cc3a526112fd0d%7C1%7C0%7C637883788858975366%7CUnknown%7CTWFpbGZsb3d8eyJWIjoiMC4wLjAwMDAiLCJQIjoiV2luMzIiLCJBTiI6Ik1haWwiLCJXVCI6Mn0%3D%7C3000%7C%7C%7C&sdata=oXWfQZsUydjy%2FLv5qBKWqvE63SpiO1GDyCbFSTAK%2BzM%3D&reserved=0)) may be used to calculate LR+ and post-test probabilities [27]. The latter two steps were not followed in this study.

Results:

In the present study, IASQ cut-offs of 1 and 2 were associated with LR+s of 5.98 and 22.42 respectively.

Discussion:

…………. As for diagnostic likelihood ratio in the present study, IASQ cutoffs of 1 and 2 were associated with LR+s of 5.98 and 22.42 respectively which are high and acceptable. While its non-linear nature is a known drawback of the diagnostic likelihood ratio or DLR (denoted by LR+ in this paper), it provides the best measure of the discriminative power of a test by summarizing both its sensitivity and specificity.  Furthermore, unlike sensitivity or specificity, DLR remains unaffected by the prevalence of the disease. The LR+ of the IASQ are also acceptable.

6. PLOS authors have the option to publish the peer review history of their article ([what does this mean?](https://journals.plos.org/plosone/s/editorial-and-peer-review-process#loc-peer-review-history)). If published, this will include your full peer review and any attached files.


If you choose “no”, your identity will remain anonymous but your review may still be made public.


**Do you want your identity to be public for this peer review?** For information about this choice, including consent withdrawal, please see our [Privacy Policy](https://www.plos.org/privacy-policy).

Reviewer #1: No

[NOTE: If reviewer comments were submitted as an attachment file, they will be attached to this email and accessible via the submission site. Please log into your account, locate the manuscript record, and check for the action link "View Attachments". If this link does not appear, there are no attachment files.]

While revising your submission, please upload your figure files to the Preflight Analysis and Conversion Engine (PACE) digital diagnostic tool, <https://pacev2.apexcovantage.com/>. PACE helps ensure that figures meet PLOS requirements. To use PACE, you must first register as a user. Registration is free. Then, login and navigate to the UPLOAD tab, where you will find detailed instructions on how to use the tool. If you encounter any issues or have any questions when using PACE, please email PLOS at [figures@plos.org](mailto:figures@plos.org). Please note that Supporting Information files do not need this step.
