# Supplementary material for: Comparing the Indian Autism Screening Questionnaire (IASQ) and the Indian Scale for Assessment of Autism (ISAA) with the Childhood Autism Rating Scale–Second Edition (CARS2) in Indian settings
Source: PLoS One. 2022 Sep 19;17(9):e0273780. doi: 10.1371/journal.pone.0273780 (PMC9484635; doi:10.1371/journal.pone.0273780)
Supplement: Attachment — Submitted filename: Reply to reviewers comments second review 05082022.docx [file pone.0273780.s004.docx]

Dr. Smita N. Deshpande

Professor

Dept. of Psychiatry, De-addiction Services,

Centre of Excellence in Mental Health,

Atal Bihari Vajpayee Institute of Medical Sciences &

Dr Ram Manohar Lohia Hospital,

Banga Bandhu Sheikh Mujib Road,

New Delhi 110001

Phone: 011 23404269, 23404363.

Email: smitadeshp@gmail.com

5^th^ August, 2022

To,

The Editor-in-Chief

Plos One.

Ref: Resubmission of our manuscript after incorporating peer review suggesitons entitled “Comparing the Indian Autism Screening Questionnaire (IASQ) and the Indian Scale for Assessment of Autism (ISAA) with the Childhood Autism Rating Scale–Second Edition (CARS2) in Indian settings” [**PONE-D-21-32345R1].**

Dear Dr.Tord Ivarsson,

Thank you for reviewing our manuscript and getting it reviewed by an expert. The respected peer reviewer appreciated our work and we have addressed his two small comments. We thank the reviewers for very encouraging and appreciative remarks.

We hope that the manuscript is appropriate for publishing. I request that a positive decision is reached soon and that this paper will be published at the earliest.

Thank you.

Yours sincerely,

Smita N Deshpande, MD

**Comments to the Author**

1. If the authors have adequately addressed your comments raised in a previous round of review and you feel that this manuscript is now acceptable for publication, you may indicate that here to bypass the “Comments to the Author” section, enter your conflict of interest statement in the “Confidential to Editor” section, and submit your "Accept" recommendation.

Reviewer #2: (No Response)

Thank you for accepting our replies to your comments.

2. Is the manuscript technically sound, and do the data support the conclusions?

The manuscript must describe a technically sound piece of scientific research with data that supports the conclusions. Experiments must have been conducted rigorously, with appropriate controls, replication, and sample sizes. The conclusions must be drawn appropriately based on the data presented.

Reviewer #2: Yes

Reply: Appreciate your comment.

3. Has the statistical analysis been performed appropriately and rigorously?

Reviewer #2: Yes

Reply: Thanks for positive comment.

4. Have the authors made all data underlying the findings in their manuscript fully available?

The [PLOS Data policy](http://www.plosone.org/static/policies.action#sharing) requires authors to make all data underlying the findings described in their manuscript fully available without restriction, with rare exception (please refer to the Data Availability Statement in the manuscript PDF file). The data should be provided as part of the manuscript or its supporting information, or deposited to a public repository. For example, in addition to summary statistics, the data points behind means, medians and variance measures should be available. If there are restrictions on publicly sharing data—e.g. participant privacy or use of data from a third party—those must be specified.

Reviewer #2: Yes

5. Is the manuscript presented in an intelligible fashion and written in standard English?

PLOS ONE does not copyedit accepted manuscripts, so the language in submitted articles must be clear, correct, and unambiguous. Any typographical or grammatical errors should be corrected at revision, so please note any specific errors here.

Reviewer #2: Yes

6. Review Comments to the Author

Reviewer #2: Thank you for inviting me to review this article. I find the study well designed and analyses excellent. Background and rationale well described, and discussion of results clear and useful. I agree with the comments of previous reviewers.
Thank you very much for your encouraging comments and appreciation.

I have only a few further comments/suggestions:

Query 1: In the "Sample" section for clarity it could be mentioned that the sample consists of 70 children with autism, and 215 children without autism, and ref to Table 1.How were the autism diagnoses ascertained? By team assessments? Child psychiatrists /pediatricians /psychologists? According to DSM-5 criteria I presume?
This information would support the quality of the sample.
And perhaps if there are data on the severity of the autism (Level 1, 2 or 3?) that could be added.

Reply1: We have added the following text: in sample section.

“The sample consisted of 70 children with autism, and 215 children without autism (Table1). The diagnosis was ascertained by CARS 2 ST and HF versions. CARS 2 diagnosis fulfills DSM V criteria.”

Query 2:In the section "Inclusion and exclusion criteria" it is stated that children with known comorbid or psychiatric disorders were excluded. Which disorders?

Reply 2: Thank you for this important observation. We added the following text in inclusion and exclusion criteria

Children with other comorbid psychiatric disorders, for example psychotic disorders, ADHD and intellectual disabilities were excluded. An asessment of illness severity was not available on most participants and therefore not analyzed.

7. PLOS authors have the option to publish the peer review history of their article ([what does this mean?](https://journals.plos.org/plosone/s/editorial-and-peer-review-process#loc-peer-review-history)). If published, this will include your full peer review and any attached files.

If you choose “no”, your identity will remain anonymous but your review may still be made public.


**Do you want your identity to be public for this peer review?** For information about this choice, including consent withdrawal, please see our [Privacy Policy](https://www.plos.org/privacy-policy).

Reviewer #2: No

[NOTE: If reviewer comments were submitted as an attachment file, they will be attached to this email and accessible via the submission site. Please log into your account, locate the manuscript record, and check for the action link "View Attachments". If this link does not appear, there are no attachment files.]

While revising your submission, please upload your figure files to the Preflight Analysis and Conversion Engine (PACE) digital diagnostic tool, <https://pacev2.apexcovantage.com/>. PACE helps ensure that figures meet PLOS requirements. To use PACE, you must first register as a user. Registration is free. Then, login and navigate to the UPLOAD tab, where you will find detailed instructions on how to use the tool. If you encounter any issues or have any questions when using PACE, please email PLOS at [figures@plos.org](mailto:figures@plos.org). Please note that Supporting Information files do not need this step.
